# Brownian Aging as One of the Mechanistic Components That Shape the Single-Channel Ionic Currents through Biological and Synthetic Membranes

**DOI:** 10.3390/membranes13110879

**Published:** 2023-11-11

**Authors:** Agata Wawrzkiewicz-Jałowiecka, Andrzej Fuliński

**Affiliations:** 1 Department of Physical Chemistry and Technology of Polymers, Silesian University of Technology, Strzody 9, 44-100 Gliwice, Poland; 2 Institute of Theoretical Physics, Jagiellonian University, Łojasiewicza 11, 30-348 Kraków, Poland

**Keywords:** ion channels, nanochannels, biomembranes, Brownian motions, aging, open-closed channel sequences, numerical simulations, channel-membrane interactions

## Abstract

Semipermeable membranes enable the separation of a given system from its environment. In biological terms, they are responsible for cells’ identity. In turn, the functioning of ion channels is crucial for the control of ionic fluxes across the membranes and, consequently, for the exchange of chemical and electrical signals. This paper presents a model and simulations of currents through ionic nanochannels in an attempt to better understand the physical mechanism(s) of open/closed (O/C) sequences, i.e., random interruptions of ionic flows through channels observed in all known biochannels and in some synthetic nanopores. We investigate whether aging, i.e., the changes in Brownian motion characteristics with the lapse of time, may be at least one of the sources of the O/C sequences (in addition to the gating machinery in biochannels). The simulations based on the approximated nanostructure of ion channels confirm this postulation. The results also show the possibility of changing the O/C characteristics through an appropriate alteration of the channel surroundings. This observation may be valuable in technical uses of nanochannels in synthetic membranes and allow for a better understanding of the reason for the differences between the biochannels’ activity in diverse biological membranes. Proposals of experimental verification of this aging O/C hypothesis are also presented.

## 1. Introduction

Brownian motion (BM) in strongly confined spaces (CBMs) has recently been gaining more and more attention [1]. One of the reasons for this interest is that these processes can be measured, and the results show that such motions are very different from standard normal and anomalous BMs. Another reason is that CBMs appear in various systems. Among them, the best known are biological ion channels being transmembrane proteins present in all living organisms [2,3] (including “quasi-live”, i.e., active viruses). Their functioning is crucial for the cells’ biology. They contribute to the semipermeability of membranes, which separate a given cell or its organelles from its surroundings by enabling rapid and selective transport of particular ion types down an electrochemical gradient [4,5]. The improper functioning of such channels often results in a cell’s dysfunction, apoptosis, or even cancerous mutations [6,7]. A less well-known, but used for some practical aims, example of CBM realization is the ionic currents through nanopores in various thin films (in biological or synthetic membranes) [8,9].

Moreover, all biological and some artificial CBMs possess one very specific observable characteristic feature, which remains an enigma: long series of currents flowing through such CBMs are not continuous (in a generalized sense) but are composed of irregular sequences of high and low values of currents, called “open” and “closed” (O/C) states of the channel.

**Figure 1:**
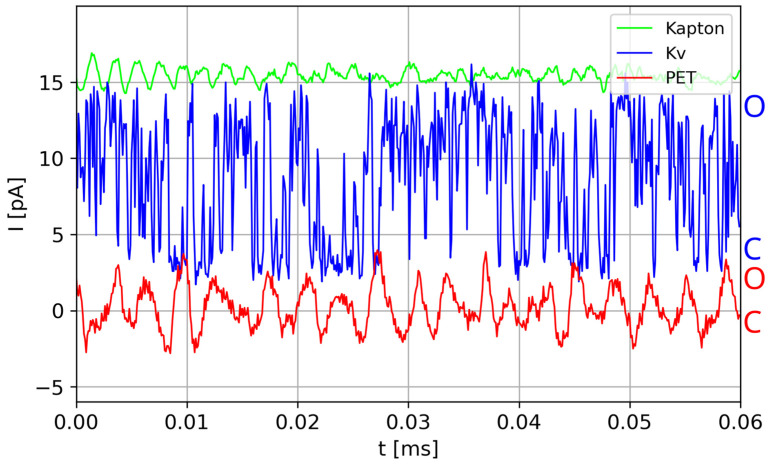
Representative samples of the single-channel currents {I(t)} flowing through a few different nanochannels: bistable (A-CH) synthetic PET channel [10] (red), bistable (A-CH) biochannel Kv [11] (blue), unimodal (NA-CH) synthetic Kapton channel [10] (green). The letter “O” indicates open state of the channel. The letter “C” indicates closed channel state. The labels A-CH and NA-CH are assigned to the traces according to the proposed aging-related O/C hypothesis.

At fixed external conditions (including concentration of ions on both sides of membrane, electric potential across the membrane, temperature, pressure, membrane strain, etc.), one can record the ionic currents flowing through a single nanopore on a real time scale using the patch clamp technique [5]. Frequently, the obtained time series of the single-nanopore currents are bistable [4,5]. Based on the current values, one can identify the functional states of the channel (open and closed) (Figure 1), according to the existing algorithms of the “event detection” [5].

The process of open/closed channel flickering, seemingly uneconomic but observed in measurements, appears to be fixed by evolution at least as early as in cyanobacteria, i.e., ca. 10^9^ years ago. According to the present models of the channel activity, the observable channel switching mainly stems from the conformational dynamics of the biological channels that results from the functional coupling of its regulatory domains with the pore–gate domains [12,13,14]. Nonetheless, even if the external conditions strongly support channel opening in the consequence of pore–gate interaction, there is a non-zero probability for channel closure [4] and vice versa. Even if the closed state is preferred, there is always a non-zero probability of a brief channel opening [4]. This suggests the presence of an additional component process, which contributes to the observed transport properties of the channel. Moreover, the mechanism of the O/C changes can become trickier and they are still not fully explained when the channel possesses no mechanic gate, which can physically block the pore, as is the case, presumably, for the BK channel [13]. In addition, the flickering behaviour also occurs for the artificial channels in the synthetic films that have no ‘true’ activation gate. Therefore, a clear picture of the complete mechanism responsible for the open/closed nanochannels’ switching at arbitrary conditions remains not fully understood.

A recent trend in research on BMs is the effect/phenomenon called aging of the process [15], i.e., the changes in some BM characteristics with the lapse of time. Again, such effects appear mostly in CBMs. The present paper proposes to investigate whether aging is one of the sources of the bistable ionic current characteristics, observed as the O/C sequences, by means of an appropriate model and simulations. Therefore, the aging hypothesis should explain the existence of brief closings of biochannels when the open state is anticipated (based on the gate’s state) and vice versa. It should also justify the presence of non-conducting states of artificial nanochannels in some polymer films, e.g., PET. These channels are supposed to enable unhampered ionic conduction in the presence of external electric potential across the membrane, but the experiments show that they exhibit O/C sequences (Figure 1). The acronyms A-CH and NA-CH used throughout this paper mean aging channel and non-aging channel.

The physical basis of such a hypothesis seems quite simple. The inner walls of (almost) all bio- and some nanopores’ contain some charges on their surface. Moreover, these pore walls are coarse, with sticking fragments of their constituents (side chains of protein residues, “dangling ends” [10] of teared polymers, etc.). Additionally, the transient pore geometry and its ability to transport ions are regulated by the interactions between the pore (including selectivity filter) and activation gate(s) in biological channels [12,13] and, further, the channel’s surroundings (biological membrane [14] or polymer chains). Particles passing through the channels, in particular, passing ions, are restrained in their motions by the spatial and energetic constraints imposed by the contemporary nanopore conformation, but also the passing ions disturb the pore walls’ state. When either the obstacles block the current or the currents “damage” any important part of the channel, the channel becomes blocked and needs time to return to its conductive form. In particular, in the case of ionic biochannels, it is commonly assumed that there are three/four ions or water molecules in the channel’s narrowest part blocking the current [2,3,12]. Our hypothesis is based on the following observations (cf. Figure 1).

When measured [10] with identical apparatus and in identical conditions, ions flow through a polymer PET (soft matter) nanochannel with charged dangling ends inside the pore, exhibiting typical O/C sequences, whereas currents flowing through a similar nanochannel made from Kapton film (hard, glassy polymer) are stationary in the wide sense. Published data [16,17,18] concerning the geometrical structures of biological and viral nanochannels together with fragmentary data, on the atomic scale, of the main parts of these channels (responsible for its conductivity and selectivity) show that in the inside of these channels, there are similar substructures as in the PET channels, i.e., sticking charged fragments of peptides forming the channel.

Therefore, we decided to investigate the molecular mechanisms of O/C sequence regulation and dysregulation, and the implications for the channel transport of ions across membranes. In this regard, we propose an appropriate CBM-based model and preform numerical simulations. We pay particular attention to the structural and physical properties of the pore spanning the entirety of the membrane. In the first step, we assume a generalized structure of the channel (described in detail in Section 2), which basically describes only an empty channel. During the simulation, the presence of charged ions inside the channel induces new changes in its structure. Taking this into account requires the introduction into the model of the effective charge(s) of the channel’s walls, being the sum of permanent structural charges inside the pore and the transient charges induced there by the flowing ion(s). Basic physics suggests that such changes will not vanish instantly after the ion departure but will require some time to heal; this is actually the core of the idea of aging, with subsequent rejuvenation of the channel.

Research on this phenomenon will shed some light on perhaps the greatest enigma in nanochannelology, i.e., the seemingly uneconomic random interruptions of ionic flows through channels observed in all known biochannels and in some synthetic nanopores. The investigation of the formulated hypothesis with numerical methods and preparation of a conceptual framework for prospective experimental research are the objectives of the present project. The outcomes of the research will be compared with existing experimental data obtained from several different channels, biological and synthetic.

Of consequence is the knowledge of so-called observables on the registered series {I(t)}, i.e., the stochastic characteristics of the investigated process. Several of them are known for eukaryotic potassium channel Kv [11]. An important ramification of the proposed project, a “side effect” of the determination of observables, is that by revealing various physical and biological details hidden in {*I(t)*}, they help to understand the working of one of most important processes necessary for the proper functioning of all live cells, including animal, plant, bacterial, or even viruses: the nanochannels present in all cells of every organism ever living on Earth. Without the proper functioning of these tiny constituents of the cell, the whole organism would not function properly.

A “value-added” aspect of this project is the possibility of the visualization of nanoscale tracks of single ions passing through the channel under various channel situations (“Open” vs. “Closed”), reactions on unexpected external disturbances that affect the geometry, charge, or other characteristic pore parameters that affect the ionic transport (cf. Ref. [19]). Such information cannot be directly found experimentally.

Is experimental verification of the aging-related O/C hypothesis possible? It seems easy to check the aging in synthetic channels; moving “dangling ends” can be stopped by introducing an appropriate gel to a channel or coating to effectively “freeze” these broken polymer fragments in various configurations but retain the ionic transport at the same time. Also, it is possible to cover the synthetic NA-CH with a functional layer that introduces some nanoparticles on the channel pore walls, which would play a role analogous to dangling ends in synthetic A-CH.

This task looks much more difficult for biochannels, but in the authors’ opinion, it is not impossible. Slowed-down or, contrarily, sped-up aging could be induced by some drugs, through the actions of external fields [19] or [20,21] changes in the environment. Namely, changing the biophysical properties of membranes (e.g., via lipid exchange techniques, cholesterol manipulation, genetic engineering, lipidation, acylation, glycosylation, or physical methods like sonication, squeezing, etc.) should indirectly influence the ion channels’ activity via protein–lipid and protein–protein interactions [14,20,21].

## 2. Materials and Methods

### 2.1. The Model

The model proposed here assumes that the motion of an ion is described as anomalous Brownian motion in a very confined space and with reset [22,23] under several internal and external fields of force: electrostatic interactions of ions with remaining ions and with both static and induced charges within the channel itself. We also consider the differences in the concentration of given ions inside and outside the cell membrane. Moreover, the model includes friction and fluctuations. Stochastic forces (noise) comprise the always-present thermal noise, i.e., random impulses from neighbour particles (e.g., membrane surroundings), according to Maxwell’s distribution and the more or less random influences of neighbour processes. The model has two main variants depending on the channel type, i.e., it can describe the ionic transport through synthetic channels and biochannels (cellular channels or viroporins). Data from [10] show that the core of PET synthetic channel contains semi-moving “dangling ends” of broken polymer chains; data from [18,20,21], related to the atomistic structures of the KcsA channel and E-viroporin of SARS-CoV-2, suggest that the inner surfaces of biochannels are coarse, both in a mechanical and electrostatic sense. Different structural characteristics are assumed for the two distinguished channel types, as discussed below.

Structure of synthetic channels is relatively simple [8,9,10]. They are composed of two connected asymmetric narrow cones, chemically etched in thin polymer foils. Most narrow part of such channels (nanopores) has radii of about one to a few nm, comparable with most narrow parts (selectivity filter) of biochannels.

**Figure 2:**
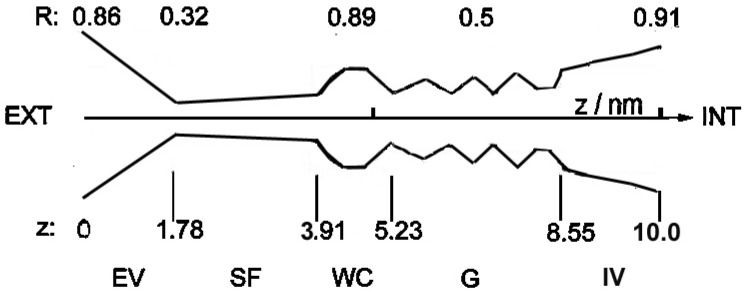
The sketch of general geometrical structure of a biological ionic nanochannel based on KcsA K^+^ channel presented in Figures in [16,17]. The similar general structure of the SARS-CoV-2 E-viroporin is discussed in [18]. Details: EV: external vestibule, SF: selectivity filter (single-file motion), WC: water cavity, G: gate, IV: internal vestibule. R: [numbers, nm] the diameter of the channel pore for its different functional parts; z: [numbers, nm] describe distance along the channel longitudinal axis from the external entrance (*z* = 0) towards the interior (*z* = 10 nm).

Schematic structure of bio-channels is shown in Figure 2 below. This generalized representation is simple but clearly represents the main structural parts of the channel proteins.

Referring to our previous work, where another model of ionic transport through channels was formulated [19], Figure 2 shows an improved version of the pore geometry. The main changes are as follows: the cylindrical shapes of all fragments are changed into more realistic conical ones (GATE). Moreover, in calculations of ions’ trajectories through the channel (cf. Section 2.2 Equations of motion), the effective friction *ρ*, earlier the same along the channel, is changed into *ρ*(*z*), different in different fragments (highest in SF). Simulations have shown that results of several other small changes are negligible.

The physical parameters of ion channels are as follows: length of about 10 nm, internal diameter of a few nm, in the narrowest part (Selectivity Filter, cf. Figure 2), radius of less than 1 nm (about 3 angstroms in diameter), and strong asymmetry along the *z*-axis. Inside the channel, ions move under very strong external electrostatic fields. It is worth noting that biochannels move randomly (through a kind of 2D Brownian motion) in semi-fluid cell (or organelle) membranes [24].

One of the most important properties in the structure of all ionic nanochannels considered here is their strong asymmetry along the longitudinal axis, together with relatively good axial symmetry. Another is that internal surfaces are coarse, both in mechanical and electrostatic sense, and consist of sticking out fragments of broken polymer chains in synthetic pores or fragments of atomic-scale details of bio- ones. These fragments are able to make limited motions and interactions with passing ions.

**Figure 3:**
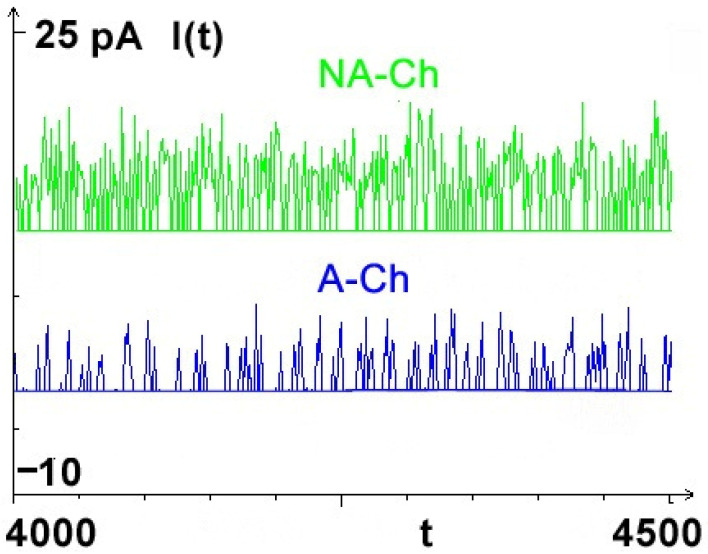
Representative samples of currents {I(t)} computed for the same simulated biochannel in two variants i.e., aging A-CH (blue) and non-aging NA-CH (green).

The crucial assumption of the model is the implementation of optional aging in the model. As said in the Introduction, aging of the channel results from the continuous influence of passing of subsequent charged ions on the structure of the channel’s walls. These changes are twofold in nature: immediate induction of charges on the near internal surface of channel’s walls, with almost immediate relaxation of these additional induced charges, and the slow accumulation of tiny injuries in deep structure of the whole channel. It is this latter effect, which, according to the assumed hypothetical model, induces the O/C transitions. The direct realization of aging in simulation is described below in Section 2.4. Differences between aging and non-aging channel’s currents are shown in Figure 3 below.

### 2.2. Equations of Motion

Let *i*, *j* denote numbers of ions, *k* denote any element of the channel (ion *i*, charge of specific place within walls, etc.), and *x_i_* denote either *z*- or *r*-coordinate of *i*-th ion. Then, equations for the motion of ions can be written in the discrete form of anomalous Brownian motion:

(1) $$x_{i}\left(t\right)=x_{i0}+W_{xt}[A_{x}\xi \left(t\right)-\rho \left(t\right)+V_{0}+\Delta c+\sum_{k}\varphi_{i,k}+\sum_{j}{bar}_{j}]$$

(2) $$\varphi_{i,k}=F_{0}q_{i}q_{k}/L_{i,k}^{2},\;L_{i,k}^{2}=\left|z_{i}^{2}+r_{i}^{2}\right|-\left|z_{k}^{2}+r_{k}^{2}\right|$$

(3) $$\rho \left(t\right)=\rho_{c}\left(t\right)+\rho_{m}\left(t\right)+\rho_{ext}$$

(4) $$\rho_{c}\left(t\right)=\rho_{0}e_{i}e_{k}{\left[y_{i0}/(1-y_{i0})\right]}^{2},\;y_{i}=r_{i}(z_{i})/R_{i}(z_{i})$$

(5) $${bar}_{j}=e_{i}e_{barj}/(dz_{j}^{2}+10.0^{-6}),\;{dz}_{j}=z_{barj}-z_{i}$$

where *W_xt_*, *A_x_*, ΔC, *F*_0_, and *ρ* are parameters for which there are no observational data, and, moreover, they scale subsequent terms in the nanometre dimension. *R(z_i_)*—radius of the channel at *z_i_*, *x_i_*_0_ = *x_i_* (*t* − *δt*); *δt*—micro time-step (usually 10–100 ns); *ρ*—effective friction, being the sum of electrostatic, mechanical, and external ones; *ρ_m_*—the deceleration by sticking-out peptide fragments (the latter being an analogy of ‘dangling ends’ in synthetic channels [10] cf. respective figures in [16,17,18]); *ρ_ext_*—any additional external disturbances; *bar*—electrostatic interactions of ion *i* with both static and induced charges within channel’s walls.

### 2.3. Model Implementation and Introduction of Aging

**Figure 4:**
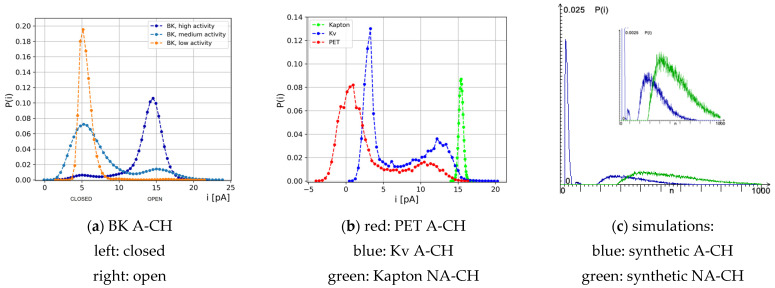
Comparison of histograms of series {I(t)} of different ionic nanochannels, biological (BK at three different levels of activation (**a**) and Kv about half-activation (**b**)—blue trace), synthetic (PET (**b**)-red and Kapton-green), and the simulated A-CH and NA-CH channels (**c**). P(i) is a probability of finding a given value of current in the analyzed series. Note characteristic bi-(A-CH) and mono-(NA-CH) modality. The experimental data are adapted from (**a**): [25] (**b**): [10,11] and re-examined.

In simulations, an individual ion changes its position *x(t)* to *x(t + δt)* (in simulations *δt* is ca. 10^−9^–10^−7^ s), where the vector x denotes the position within the channel (described in Figure 2 as (*R,z*)). The registered ionic currents {I(t)} (in pA) passing through channel are calculated as the number of ions which enter the channel pore via one of the vestibules (cf. Figure 4) and leave the channel pore through the other one during the time step Δ*t* = 0.0001 s, the latter being the typical time window of measuring devices.

Aging is realized in the computer program by so-called micro-stairs: changes at every micro time-step *nδt* (usually 10–100 ns) of the velocity of the ion. This is implemented directly by the changes in the parameter *W_xt_* (cf. Section 2.2 Methods/2.2 Equations of motion). In open state of the channel *W_xt_* decreases; in closed state, it increases by micro-step *δW_xt_* (different in O- and C-state). At every subsequent step, the micro-local velocity of the ion varies due to fluctuations in all parameters in equations of motion. When the value of the *W_xt_* reaches the prescribed (though also fluctuating a little) limiting value, channel changes its microstate from open to closed, or vice versa, and begins its march along the staircase in the inverse direction.

### 2.4. Fluctuations

To keep with the physical reality in which biochannels work, all parameters, both those written explicitly in Equations (1)–(5) and others used throughout modelling/computations, are allowed for higher or lower fluctuations. These, though, usually do not influence the general interpretation of the results or change the details, in particular, the shapes of macro-trajectories of registered series of currents {I(t)}, as presented in Figure 1.

In particular, even the height of “steps” in the “staircase”, where micro-aging/recovery occurs, fluctuates: micro-step = *nδt*, with *n* ∈ [1, *nmax*] chosen randomly. Usually, *nmax* = 5 was used.

## 3. Results

The motion of an ion is described in our model as anomalous Brownian motion in a very confined space and with reset [22,23]. Ions move due to the actions of several natural, external and internal force fields (built-in and induced ones): electrostatic, chemical, friction, and stochastic; the latter is known as ‘noise’. According to the obtained results, the introduction of aging in the model allowed us to reflect a typical bistable single-channel current signal (Figure 3). 

Considering the distributions of single-channel currents, the majority of ion channels in biological membranes exhibit bimodal characteristics as presented in Figure 4a,b (for the Kv channel), whereas, in polymer nanopores, both bimodal and unimodal histograms of currents can be observed, depending on the structural and physicochemical properties of nanopore walls and internal interactions within the polymer film (please compare the results for nanopores in PET and Kapton membranes in Figure 4b). The results obtained by the simulation of synthetic nanopores according to the proposed model allow us to reproduce both types of distributions. When the aging effect is activated, the *I(t)* series is bistable, whereas, when micro-stairs are deactivated, there is no O/C series, and P(i) is unimodal. This is illustrated in Figure 4c.

**Figure 5:**
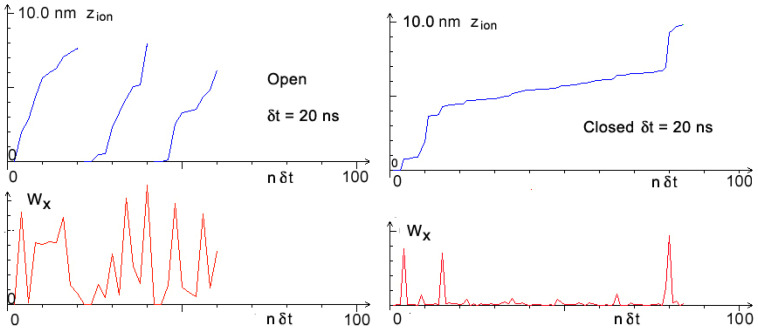
Visualization of dependence of microscale velocity of an ion steering parameter *W_x_*. Nanotracks of an ion inside bio nanochannel (upper part) and the corresponding values of *W_x_* (lower part). Visible are accelerations and decelerations depending on changing values of *W_x_*. Velocity of ion inside Open channel (left part) is about five times higher than velocity of ion inside Closed channel (right part).

Because micro-motions seem to play the deciding role in aging, we simulated how these unobservable nanoscale tracks of single ion velocity—mentioned in the Introduction—depend on *W_xt_*. The result is presented in Figure 5.

Figure 5 also illustrates the role of microfluctuations—the unexpected appearance of one stronger impulse in the steering parameter *W_xt_*, resulting from either random fluctuations of some physical parameters or from, e.g., the action of an external physical field [25]. At this point, the physical interpretation of *W_xt_* and its role and place in the computer program, as described in more detail in the Methods, require some explanation.

This one parameter comprises several different effects. Formally, in the ion’s equations of motion, Equations (1)–(5), it describes the effective velocity of the ion (or its inverse: effective friction) and its fluctuations, but the latter also depends on fluctuations in several other parameters in equations of motion. Instead of explicitly introducing these different fluctuations, one obtains the same effect through fluctuations in this single effective “velocity/friction”. Living bodies and living cells are exposed to many different unexpected external or internal actions; the only real possibility in simulations is to treat these as generalized random fluctuations. And a computer draws everything.

## 4. Discussion

In this work, we approached the problem of the possible mechanism of open/closed ion channel switching through the formulation and simulation of an ionic transport model, which takes into account key factors affecting the motion of ions, including the pore dynamics, that shape the electrical potential and chemical gradients across the biological and some synthetic membranes. The results presented here prove that the micro-aging process results in the appearance of irregular sequences of O/C states of the channel in the macro-trajectories of registered series of currents {*I(t)*} (Figure 3 and Figure 4c). Excluding micro-aging leads to the loss of one of the groups of currents, i.e., this results in maintaining the channel in the same initial functional state: permanent opening or closure.

The main result of the simulations presented in this paper is as follows: aging is at least one of the factors causing the appearance of enigmatic O/C sequences. When micro-stairs are activated, their presence somehow influences the channel’s macrostate, resulting in a series of recorded high and low values of *I*(*t = n*Δ*t*). In these terms, the distribution *P(I)* takes the bimodal form (Figure 4c). Our results provide a reasonable explanation of the bimodal characteristics of empirical data, particularly for the nanochannels without advanced conformational gating machinery, i.e., synthetic nanopores or biochannels of the relatively simple structure and/or gating machinery that enables only one leading stable (physically open or closed) conformation in fixed experimental conditions.

The other result worth mentioning, though not discussed here in detail, is that it is possible to change the O/C behaviour through appropriate changes in the environment in which the channel is immersed. This result might be of practical value, in particular in the technical applications of synthetic nanochannels. Based on these fragmentary simulated data, propositions of the direct experimental verification of the aging–O/C hypothesis, realistic manipulations of O/C sequences, and further investigations in this direction are suggested in more detail at the end of Section 1.

Considering the limitations of this study, first, a relatively simple representation of channel structures was used in our model. It is to be noted that the shape of the biochannel (shown in Figure 2) is only the generalized author’s model, which is based on relatively scarce published atomic-scale structural data, on equally scarce pictures (some sketchy) of biochannels, and in literature sketches or symbolic representations of this type of data. Even if in the literature and databases, like Protein Data Bank, one can encounter more detailed pictures of channels’ structures, they are frequently based on crystallographic studies, which are performed in conditions extremely far from physiology. From this perspective, even full-atom models are only a rough approximation of the real ever-fluctuating channel structures surrounded by the lipid environment. Therefore, our representation of channel structure should not be treated as reality but as a reasonable basis for simulations only. This generalized structure can correspond to the wide class of cellular ion channels but not only this. We are convinced that it can also be adapted for the description of ionic transport through viroporins.

There are almost no empirical structural data for viroporins. The results presented in ref. [15] suggest that the geometrical structure of the SARS-CoV E channel is almost identical to that of other biochannels. However, the picture presented in [18] suggests that this channel is bent near the selectivity filter. Moreover, the structure presented there is not an atomistic structure of the channel itself “in situ” but is reconstructed from the known atomic-scale structure of one E-protein subunit, of which the pentametric E-channel is built. For instance, data in [20,21] suggest that the ions inside are in contact not only with the appropriate E-protein but also with the membrane in which the viroporin penetrates. How all these differences influence the behaviour of an ionic current flowing though such a channel can, at present, only be a matter of speculation. In the present author’s opinion, these differences might be the source of viroporins’ diminished selectivity [26,27], in particular for the almost nonselective SARS-CoV E-channel [18].

Coming back to the main focus of this work and the obtained results, the fluctuations that appear in the proposed model can represent different biological effects. Thermal oscillations of the pore–gate domains and the everchanging effects of the membrane that eternally affect the actual pore architecture are collectively mimicked in the model through microfluctuations. Of course, in a real system, other processes can occur that exert functional effects on the channel functioning, like channel dewetting, randomly entering the selectivity filter vi a water molecule. In reality, large-scale conformational changes in the channel structure also occur through mechanical closing/opening/inactivation that requires translations, rotations, and/or tilts of helices forming the biochannels. Here, we only track the changes in currents resulting from aging at fixed channel macroconformations (which in a real system, results from the SF-pore–gate(s) activation). These processes are not considered in our model and can serve as inspiration for the further development of our investigations.

## 5. Conclusions

The results presented in this paper indicate that the introduction of Brownian aging in the process of the ions’ motion through the nanochannel pores leads to the bistable characteristics of the resulting currents, which can be further identified as the open and closed channel states. These inferences may be especially valuable for understanding the nature of the non-conducting states in some membranes as well as some ‘unusual’ states of biochannels, like brief closings of the fully activated channels. In this paper, we also proposed some possible ways for the experimental verification of the aging-O/C hypothesis.

## Data Availability

All data are taken from cited references. The source code of the computer program was written by the present author and can be obtained on reasonable request.
